# Prevalence of depression, anxiety, and stress and associated reasons among Iranian primary healthcare workers: a mixed method study

**DOI:** 10.1186/s12875-024-02268-w

**Published:** 2024-01-26

**Authors:** Edris Kakemam, Katayoun Maassoumi, Somayeh Azimi, Madineh Abbasi, Fateme Tahmasbi, Mahasti Alizadeh

**Affiliations:** 1 Social Determinants of Health Research Center, Health Management and Safety Promotion Research Institute, Tabriz University of Medical Sciences, Tabriz, Iran; 2 Department of Community Medicine, Faculty of Medicine, Tabriz University of Medical Sciences, Tabriz, Iran; 3 Department of Health Education & Promotion, Tabriz University of Medical Sciences, Tabriz, Iran; 4 Infectious and Tropical Diseases Research Center, Tabriz University of Medical sciences, Tabriz, Iran

**Keywords:** Mental health, Primary health care, Anxiety, Stress, Depression

## Abstract

**Background:**

Different mental disorders may be associated with many work-related factors to which primary health care workers (PHCWs) are exposed. The current research aims to measure the rates of depression, anxiety, and stress among PHCWs, and their associated causes in primary health care (PHC) settings.

**Methods:**

An explanatory sequential mixed methods design was employed in this research from January 2021 to January 2022 in Tabriz, Iran’s PHC centers. First, this study followed an online-based cross-sectional survey using a self-reported questionnaire. The Depression, Anxiety and Stress Scale-21 Items (DASS-21) and questions on demographic and work-related characteristics were completed by 303 frontline PHCWs during the quantitative phase. In the qualitative phase, a semi-structured interview was held with 12 PHCWs who had the highest level of depression, anxiety, and stress to identify the reasons and sources of mental health prevalence. Quantitative data were analyzed using descriptive statistics via SPSS-26. A content analysis was performed to analyze qualitative data.

**Results:**

The results showed that self-reported stress, anxiety, and depression had a prevalence of 40.3%, 42.9%, and 42.6%, respectively. Symptoms of at least one mental disorder were experienced by 54% of respondents, while 28% had all three. Major sources of stress, anxiety, and depression among PHCWs were working environment conditions, organizational policies, job-related reasons, and interpersonal relations.

**Conclusions:**

The results of current study indicated that PHCWs experienced high levels of depression, anxiety, and stress. The main factors and reasons that contributed to these mental health issues among PHCWs were work environment conditions, organizational policies, job-related reasons and interpersonal relations. Therefore, interventions should be implemented to promote mental health of PHCWs. This can include measures such as psychological screening, supportive care, workload management, flexible scheduling, and access to mental health resources. Additionally, training programs can be implemented to enhance resilience and coping skills among healthcare professionals.

## Background

The foundation of all health services is primary health care (PHC), which includes prevention, treatment, management and rehabilitation services [1]. PHC has a distinct role in global health systems to achieve universal health coverage (UHC) and improve the health of populations [2]. Moreover, Infection control, risk management, and public health response all depend on primary healthcare providers (PHCWs), who also provide medical care for ongoing or urgent health issues [3]. Therefore, a PHC system requires a skilled, effective, and motivated personnel to deliver high-quality care. However, structural reforms in the health system have resulted in excessive workload, increased turnover rates and job dissatisfaction, which may lead to mental health disorders among health workers [4–6]. Healthcare workers encounter a range of stressors in their daily work. Heavy workloads and workforce shortages place immense pressure on healthcare professionals, leading to increased stress levels and potential burnout. Team conflict and lack of role clarity can further contribute to stress and job dissatisfaction. Inadequate time to perform work tasks can create a sense of overwhelm and hinder the delivery of quality care. Low support from managers and ineffective task distribution can exacerbate stress levels and negatively impact mental well-being. Additionally, witnessing patient suffering and death can be emotionally challenging and traumatic for healthcare workers [3, 7, 8]. The last two decades have seen a substantial increase in study interest worldwide about mental health issues among PHCWs [7, 9–11].

Common health disorders like depression, anxiety, and stress have a significant impact on the global burden of disease [12]. It has been estimated that 4.4% of people are affected by depression and 3.6% by anxiety disorders globally [13]. Among all professions, PHCWs are among the most susceptible to mental health issues. For instance, a study among Malaysian PHCWs reported that 19.7%, 15.2%, and 2.8% of them suffered from depression, anxiety, and stress, respectively [14]. Another study from Turkey found that 10/9%, 14.8%, and 5.0% of PHCWs had depression, anxiety, and stress, respectively [9]. According to data from Iran, more than 50% of healthcare professionals working in hospitals and PHC facilities suffer from mental illnesses [15].

Several studies have examined mental health and its risk factors among primary healthcare workers in different countries such as Turkey [9], Malaysia [11, 14], Brazil [7], Oman [16], India [10], and Australia [17]. However, there is little data in Iran, particularly with reference to stress, anxiety, and depressive disorders. The paper aims to address the gap in the literature regarding the mental health of primary healthcare workers in Iran. While studies on mental health among healthcare workers have been conducted in various countries, there is a need for context-specific research to understand the unique challenges faced by primary healthcare workers in Iran. Factors such as cultural influences, healthcare system structures, and societal norms can contribute to distinct experiences of mental health issues among this population.

By employing a mixed-method approach, the researchers aim to obtain a comprehensive understanding of the prevalence of depression, anxiety, and stress among Iranian primary healthcare workers. The quantitative component of the study will involve administering standardized questionnaires to assess the prevalence and severity of these mental health conditions. This will provide quantitative data that can be analyzed to determine the extent of the problem and identify potential risk factors. Additionally, the qualitative component of the study will involve conducting interviews or focus groups with primary healthcare workers. This qualitative data will allow researchers to delve deeper into the experiences, perceptions, and underlying reasons for the prevalence of mental health issues. It will provide valuable insights into the unique challenges faced by Iranian PHCWs and help identify potential strategies for prevention, intervention, and support. The findings of this research are expected to contribute to the existing body of knowledge on the mental well-being of healthcare professionals, particularly within the Iranian context.

## Methods

### Study design

We used explanatory sequential mixed methods design to improve the validity and credibility of the study results. Quantitative approaches were carried out first, followed by qualitative ones in that order. We used qualitative data to complement the quantitative data, assuring complementarity and giving PHCWs a complete and in-depth understanding of the reasons and sources of excessive stress, anxiety, and depression [18].

### Quantitative phase

An online cross-sectional survey was carried out among health professionals employed by PHC facilities in East Azerbaijan, a province in northwest Iran, from January 2021 to January 2022.

#### Population and sample

The target population included all health workers in PHC centers, including physicians, midwives, health care providers, trained community health workers (Behvarz in Persian), environmental and mental health experts, technicians, and administrative staff. They were working in 30 urban health centers and 63 rural health centers and health houses. Inclusion criteria were all PHCWs who had worked in the PHC for more than one year in urban or rural areas and had direct or indirect contact with patients and agreed to participate in this study. Newly hired health workers and part-time health workers and employees who had a history of physical or mental illness that affects depression and anxiety were excluded. We calculated the sample size to estimate a population proportion with a specified absolute precision. Preliminary information to calculate the effect size was obtained from the study of Khademian et al., [19] considering the confidence limits of 95%, the test power of 80% and using G*Power software for the study, the optimal sample size by calculating 20% drop. The final sample size was at least 300 subjects. We invited all health centers that provided primary health care in urban and rural to take part in the survey. The online questionnaire was sent to the centers that agreed to participate in the current study. As no specific list of people working in PHC centers existed in the district, we used the convenience sampling method.

#### Instrument

We used a self-administered survey to collect data, which included six demographic questions, five questions on working conditions, and the short form Depression, Anxiety and Stress Scale-21 Items (DASS-21) [20, 21]. DASS-21 is the new brief version of DASS-42 that was developed by Lovibond and Lovibond (1995) that measure the negative emotional states of depression, anxiety, and stress thorough 21 items (7 items per subscale), and the final score for each subscale is obtained by summing the scores of the relevant questions. The shorter version of DASS (DASS-21) was established in subsequent research. Each item is scored from 0 (does not apply to me) to 3 (completely applies to me) and since the questionnaire is a shortened form of the main scale of 42 items, the final score of each subscale is doubled in the calculation.

Table 1 shows the cut-off values for each sub-scale. Cronbach’s Alpha coefficients of the original version were calculated by Lovibond and Lovibond in 1995 as 0.91, 0.84, and 0.90 for depression, anxiety, and stress, respectively [22]. The DASS21 was translated from English into Farsi according to a multi-step process, including forward and back-translations and steps to ensure the conceptual equivalence of the measures and reviewed by a group of health professionals and research workers for appropriateness of language and cultural idioms and back-translated to English for verification [23]. According to the Iranian validity and reliability study among nurses, the Cronbach’s alphas for the DASS-21 subscales were 0.90 for depression, 0.87 for anxiety, and 0.89 for stress [24]. We created a variable of combined mental disorders by adding all three categorical variables stress, anxiety, and depression. The number of disorders ranged from 0 (no disorder) to 3 (all three disorders). The cut-points for the indicators of stress, anxiety and depression indices were above 14, 7, and 9 respectively (Table 1) [20].

**Table 1:** The scores of each subscale of depression, anxiety and stress

Severity	Depression	Anxiety	Stress
Normal	0–9	0–7	0–14
Mild	10–13	8–9	15–18
Moderate	14–20	10–14	19–25
Severe	21–27	15–19	26–33
Very Severe	+ 28	+ 20	+ 33

#### Data collection

Data collection carried out through an online questionnaire on the Survey Porsline platform. Before conducting the survey, an online meeting was held with all managers of PHC facilities to explain the objectives of the study. The regional health center sent weekly reminders to all PHCs in urban and rural areas to improve the response rate among participants. The first question in the survey was informed consent, and only participants who agreed could proceed with the online survey.

#### Statistical analysis

We analysed the quantitative data using the SPSS version 26.0. All data were summarized and described using descriptive statistics such as mean, standard deviation, number, and percentage.

### Qualitative phase

We implemented an exploratory descriptive design in the qualitative phase of the study.

#### Sampling method

For the qualitative phase, 12 individuals with symptoms of three mental disorders together were purposefully selected from the quantitative phase. We determined the sample size according to saturation principles that is the most common guiding principle for assessing the adequacy of purposive samples in qualitative research [25].

#### Data collection

Data was collected through in-depth individual interviews using unstructured and changeable questions. We asked an open-ended question: “What, in your opinion, are the causes of depression, anxiety, and stress in your job?” and they shared their personal experiences on the topic with us. We asked follow-up questions during the interview based on the interview process to obtain more detailed experiences. The sampling ended when the data reached saturation and our data were saturated by the twelfth interview. Each interview lasted between 25 and 50 min and done at the employees’ places of employment in Persian.

#### Data management and analysis

At the end of each interview, we reviewed the key points raised with interviewers. The recorded interviews were listened to several times and then transcribed verbatim after each interview. The transcripts were manually coded and grouped into categories to explore the initial themes. Data collection and analysis were conducted simultaneously. The analysis of the data was conducted using transcripts in Farsi (the native language of participants) by the first author. Data was analyzed using content analysis, for the identification of recurring themes by two researchers independently (E.K and KM). Transcripts were read several times and coded, and emergent themes were identified. The themes from the raw data were identified and summarized.

#### Trustworthiness

To assess the quality of the study findings, we applied Lincoln and Guba’s trustworthiness criteria [26]. During the study’s process, the data collection and processing was observed and monitored by a qualitative research specialist, and the data analysis was performed by two researchers independently. The participants of the study included PHCWs varying in age, level of education, working status, and working experience to help identify different views and concepts and heighten credibility. The data collection, data analysis, and theory generation process was audited, which improves the study’s dependability. The results were shared with interviewers who were asked to confirm whether they accurately reflected their experiences, resulting in better transferability within the study.

## Results

### Demographic characteristics of sample

The questionnaire was completed online by 303 PHCWs from 30 urban health centers and 63 rural health centers and health houses. Table 2 shows that most of the participants were older than 30 years (79.9%, mean age of the whole sample was 38.4 ± 8.4), female health workers (71.0%), with academic training (83.3%), married and living with a spouse (74.9%), having children (69.3%) residing in urban areas (79.2%) and had more than 10 years of PHC work experience (59.1%, mean work experience of the whole sample was 13.3 ± 8.9). Moreover, 23.4% of respondents had a history of depression and anxiety and 9.6% of respondents had a chronic disease as an underlying condition.

**Table 2:** Demographic and selected features of participants (*n* = 303)

Variables	Number	Percentage
**Gender**		
*Male*	88	29.0
*Female*	215	71.0
**Marital status**		
*Single/divorced/widow*	76	25.1
*Married*	227	74.9
**Age** (year)		
*21–30*	61	20.1
*31–40*	113	37.3
*> 40*	129	42.6
**Occupation**		
*Physicians/Midwives/ health care provider*	121	39.9
Other*	182	60.1
**Education**		
*Vocational training*	51	16.8
*Associate degree*	32	10.6
*Bachelor’s degree*	135	44.5
*Postgraduate (Master, MD, PhD)*	85	28.1
**Years at the PHC (year)**		
*< 10*	124	40.9
*> 10*	179	59.1
**Having children**		
*No*	94	31.0
*Yes*	209	69.0
**Residence**		
*Rural*	63	20.8
*Urban*	240	79.2
**Taking medication**		
*Yes*	45	14.8
*No*	255	84.2
**History of depression and anxiety**		
*Yes*	71	23.4
*No*	229	75.6
**Chronic diseases**		
*Yes*	29	9.6
*No*	271	89.4

* Environmental and mental health experts, technician, and administrative staff

### Status of stress, anxiety and depression

**Fig. 1:**
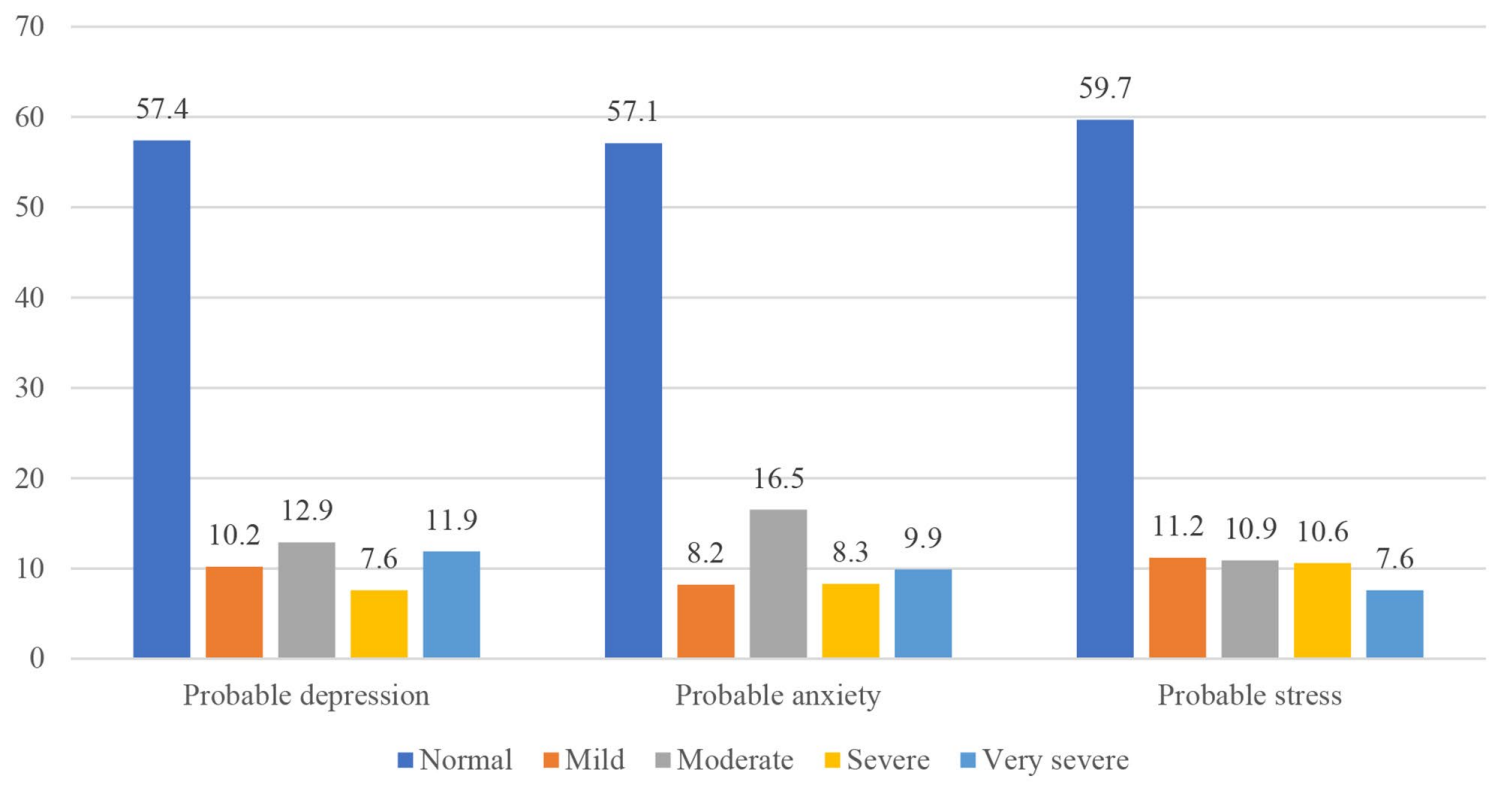
Levels of depression, anxiety, and stress

As shown in Table 3 and Fig. 1, the rates of depression, anxiety, and stress symptoms were 42.6%, 42.9%, and 40.3%, respectively. At least one mental disorder was present in 54.1% of the participants of study.

**Table 3:** The prevalence of depression, anxiety, and stress among the participants

	Number	Percentage	Mean (SD)
**Probable depression**			10.71 (10.87)
*Normal*	174	57.4	
*Mild*	31	10.2	
*Moderate*	39	12.9	
*Severe*	23	7.6	
*Very severe*	36	11.9	
**Probable anxiety**			7.97 (8.19)
*Normal*	173	57.1	
*Mild*	25	8.2	
*Moderate*	50	16.5	
*Severe*	25	8.3	
*Very severe*	30	9.9	
**Probable stress**			13.92 (10.83)
*Normal*	181	59.7	
*Mild*	34	11.2	
*Moderate*	33	10.9	
*Severe*	32	10.6	
*Very severe*	23	7.6	
**Number of disorders (**Depression, anxiety, and stress**)**			
Not any	139	45.9	
1 disorder	36	11.9	
2 disorder	43	14.2	
All 3 disorders	85	28.1	

### Qualitative results

Of the 12 people who participated in the interview, 7 were women and 5 were men. They consisted of 6 midwives and health care provider, 4 physicians, 2 mental health experts.

### The reasons and sources of high prevalence of depression, anxiety, and stress

We identified four main themes that explain the high prevalence of depression, anxiety, and stress among PHCWs: working environment, organizational policies, job-related reasons, and interpersonal relations (see Table 4).

**Table 4:** The reasons and sources of high prevalence of depression, anxiety, and stress among PHCWs

Main them	Sub-theme
**Working environment**	• *Inappropriate working condition* • *Inadequate resources*
**Organizational policies**	• *Low salary and benefits* • *Staff shortages* • *Lack of recognition and promotion prospects* • *Lack of job security*
**Job-related reasons**	• *Excessive workloads* • *Work-life imbalance* • *Role ambiguity*
**Interpersonal relationships**	• *Disrespectful behavior of patients and colleagues* • *Lack of management support* • *Poor communication skills*

#### Working environment

The participants’ statements on reasons and sources of high prevalence of depression, anxiety and stress extracted “working environment” as the first category, which included inappropriate working condition and inadequate resources. Many participants reported that the working conditions in PHC centers are inappropriate. For instance, one of the participants said:

*“Our workplace buildings are worn out; they do not have proper ventilation. Due to the limited physical space, it is always crowded here.”* (P3- Midwife).

The interviewees also stated that Iran’s PHC is constantly facing a shortage of financial and physical resources, which is one of the sources of high prevalence of depression, anxiety, and stress for PHCWs.

“*When the budgets are not enough and we don’t always have enough equipment and medicine to respond to the patient, we get a lot of worry and stress.*” (P5- physician).

#### Organizational policies

Organizational policies were the second main theme that emerged from data analysis. This theme includes four subthemes: low salary and benefits, staff shortages, lack of recognition and promotion prospects, and lack of job security. The participants in the PHC sector reported that they were paid less than the hospital care staff. Even within the PHC sector, some staff received higher salaries. They were unhappy with their monthly salary and benefits. Therefore, financial issues seem to be one of the main causes of depression, anxiety, and stress among them.

*“We get much lower salary than the hospital staff, even some private PHC staff get less salary than us”* (P1- health care provider).

A common source of stress mentioned by most participants was the staff shortages in the centers. Due to the insufficient staff, they had to do a lot of different tasks during the day.

*“I have to see too many patients as I am the sole healthcare professional at this facility, and I am also in charge of the management and planning of the center.”* (P12- physician- P4- physician).

The lack of promotion opportunities was another source of depression, anxiety, and stress cited by the interviewees. Despite having university degrees, they still had not advanced in their careers in various ways.

*“There is no promotion program here. I have been working as a midwife for 12 years, while I have continued until my master’s degree, but I have not made any career progress.”* (P10- Midwife).

Most of the staff in Iran’s PHC are temporary and they have annual contracts. The uncertainty of their jobs is one of the factors that contribute to depression, anxiety, and stress.

*“We are contract employees, and they sign a contract with us every year. Every year they may say that they want to downsize.”* (P9- Midwife- P12- mental health experts).

#### Job-related reasons

Job-related reasons were another main theme that emerged from this study, which consisted of three subcategories: Excessive workload, work-life imbalance, and role ambiguity. The PHC centers are faced with staff shortage, which results in an excessive workload for the employees. They must handle multiple responsibilities at once. This situation causes stress and anxiety.

*“I have to do many things during the day, such as providing services to patients, doing administrative affairs, and requesting medication and equipment, etc.”* (P4- physician).

*“There is no clear description of duties and responsibilities for employees. Every moment we should expect to do something new…”* (P3- Midwife and P7- health care provider).

#### Interpersonal relations

The results of data analysis revealed that interpersonal relationship problems among PHCWs with colleagues and patients also caused depression, anxiety and stress in the participants of this study. The interviewers mentioned communication issues and disrespectful behaviors that affected working relationships and impacted patient care. This main category consists of three subcategories such as disrespectful behavior of patients and colleagues, poor communication skills, and lack of management support.

One of the midwives stated that *“Most of the time, patients act rudely with the staff. They do not show any respect to the medical staff.“(*P6- midwife).

One of the challenges that employees in PHC face is the absence of support and appreciation from the managers of the organization.

*“When there is a problem, we do not receive any support from the administrators of the unit and the university”* (P8- health care provider).

## Discussion

To our knowledge, this is the first mixed study that determined the prevalence of depression, anxiety, and stress and the causes associated with high prevalence among PHCWs in Iran. Our study showed that most PHCWs in Iran experienced at least one mental disorder (54%). The rates of depression, anxiety and stress were 42.6%, 42.9% and 40.3% respectively.

Overall, the findings of the study demonstrated that PHCWs experienced higher rates of depression, anxiety and stress than other countries. For example, in Turkey, researchers carried out a cross-section study between April and June 2019, also using the DASS 21, and reported lower rates of depression, anxiety and stress: 10.9%, 14.8%, and 5.0%, respectively [9]. A recent survey in Australia found that 39.6% of nurses working in PHC have experienced symptoms of depression, anxiety, or stress [17]. Findings from a research carried out on 1520 PHCWs in Sepang, Malaysia showed that 30.3%, 36.8%, and 10.5% of staff suffered from depression, anxiety, and stress, respectively [11]. Furthermore, findings of a previous study in Oman revealed that 18.1% of the PHCWs suffer from symptoms of depression [16]. These discrepancies between our study’s findings and those of prior research might be the consequence of variations in organizational factors such as management/leadership styles, organizational climate, organizational policies and cultural issues in primary healthcare systems. One possible explanation for these dissimilarities in prevalence is differences in the socioeconomic status of participants, organizational policies and dominant organizational culture in the workplace. Moreover, the inconsistent findings could be explained by difference in patients’ expectations, as patients’ expectations can lead to a high levels of stress and anxiety.

Qualitative findings revealed that the main reasons of high levels of depression, anxiety and stress among Iranian PHCWs were working environment, organizational policies, job-related factors, and interpersonal relations factors.

***Working environment factors*** such as unsuitable working conditions and insufficient resources appear to be chief causes of depression, anxiety, and stress for Iranian PHCWs. Previous studies have associated unhealthy and improper nursing work environments to poor mental health, especially anxiety and insomnia [27, 28]. The World Health Organization has suggested workplace health promotion as one strategy to reduce and prevent job stress [29]. Accordance with findings of studies carried out in Ghana [30] and Iran [31] that unavailable of supplies and equipment induces stress in nurses, our study also found that PHCWs face challenges due to insufficient supplies and equipment. Therefore, the PHCWs who struggle to get the required supplies and equipment to work with feel the impact.

Our findings showed that ***organizational policies*** such as low salary and benefits, inadequate staff, lack of recognition and promotion prospects, improper policies and regulations, and lack of job security were other significant predictors of depression, anxiety, and stress among study participants. Organizational policies lead to a change in the nature of work for various workers and eventually their layoff or transfer. Lack of benefits and rewards is a growing source of employee frustration. Staff shortages have been identified as a cause of stress among other health workers such as nurses [30, 32]. A systematic review demonstrated that the lack of motivations in payment systems used in the Iranian PHC and the dominance of salaries and fees over payments for services are weaknesses of payment in the Iranian PHC system [33]. Previous studies have also confirmed that PHC is currently under-resourced [34, 35]. The PHC system in Iran faces fundamental weaknesses, inappropriate management of human resources and an imbalance between the supply and demand for health workers [33]. Johan et al. added that the shortage of nursing staff exacerbates the workload problem, leading to additional amounts of work [36]. Doshmangir et al., found that the lack of a working system for continuing professional development and growth prospects for staff in PHC demotivated them [34]. Therefore, it seems necessary to modify organizational policies in order to improve the mental health of employees in PHC.

The study demonstrated that Iranian PHCWs experience depression, anxiety, and stress due to ***job-related factors*** such as excessive workloads, work-life imbalance, and role ambiguity. The workload also increased the conflict between their work and personal roles [37]. Likewise, studies conducted in advanced care settings have shown that workload negatively affects the work-life balance and mental health of health workers [8]. These results agree with another similar study that found that nurses in PHCs in Saudi Arabia with high or average role conflict and ambiguity had more stress than those with low role conflict and ambiguity [38]. A heavy workload causes job stress [39], reduces job satisfaction [40] and quality of work life perception [41], and increases the turnover rate.

The findings also revealed that ***interpersonal relationship*** factors such as poor communication skills, lack of managerial support and rude behaviors from coworkers caused depression, anxiety, and stress among participants. Likewise, Ackah and Kwashie reported that inadequate communication and rude behaviors pose significant stress to hospital nurses [30]. These findings are similar with prior studies in America, Australia and Sweden that showed that low supervisor support increases the risk of mental health problems, especially of burnout, depressive, and anxiety disorders [37, 42, 43]. Managers deal directly with personnel and are in a position to play as role models in the organization [44, 45]. Consequently, evidence have approved relations between supportive leadership behavior and positive mental health outcomes for employees [46–48] including reduced risk of sick leave [49]. A prior review suggested that leadership behaviors, attitudes and social modelling had an impact on worker absence with manager behavior playing an crucial role in determining career outcomes of stressed and absent staff [50]. Developing good personal relationship at workplace is essential for the prevention of depression, anxiety, and stress among PHCWs. Education, training, and promoting teamwork help improve cooperation among PHCWs and clinical professionals.

### Strengths and limitations of the study

This study has two main strengths. Firstly, this paper employs an explanatory sequential mixed methods approach, which is a strength. This design allows for the collection and integration of both quantitative and qualitative data, providing a more comprehensive understanding of the research topic. Secondly, the paper utilizes the short form Depression, Anxiety, and Stress Scale-21 Items (DASS-21) to measure mental health symptoms. This instrument has been widely used and validated, providing a standardized and reliable measure of depression, anxiety, and stress. The use of a validated tool enhances the accuracy and comparability of the results. Despite the aforementioned strengths, there were several limitations which restricts the methodological vigor. Initially, the paper uses convenience sampling for the quantitative phase, which introduces the possibility of sampling bias. Convenience sampling relies on individuals who are easily accessible or readily available, which may not represent the entire population of primary healthcare workers in West Azerbaijan. The findings may not be generalizable beyond the specific sample and may not reflect the experiences of all healthcare professionals. Moreover, the study relies on self-reported data through online questionnaires and interviews. Self-report measures are subject to response biases, including social desirability bias or recall bias. Participants may underreport or overreport their symptoms or experiences, which could affect the reliability accuracy of the findings. The potential for bias should be acknowledged and considered when interpreting the results. In addition, the study lacks a control group, which limits the ability to establish causal relationships. Without a comparison group, it is challenging to determine whether the observed prevalence rates of mental disorders are specific to primary healthcare workers or if they are comparable to the general population. A control group would provide a baseline for comparison and strengthen causal inferences.

## Conclusion and recommendations

This study revealed that most PHCWs suffered from high levels of depression, anxiety, and stress owing to the work environment condition, organizational policies, job-related factors and interpersonal relations. The findings of our study highlight the need to recognize the mental status of PHCWs, whose vulnerability is often overlooked, in order to provide the adequate and improve the quality of healthcare. Therefore, primary healthcare managers should develop better depression, anxiety, and stress psychological screening and supportive care programs to enhance PHCWs’ work quality and psychological well-being. To reduce depression, anxiety, and stress, managers should focus on interventions tailored according to identified causes and sources of high prevalence of depression, anxiety, and stress.

## Data Availability

The datasets used and/or analysed during the current study are available from the corresponding author on reasonable request.

## Abbreviations

PHCWs: Primary health care workers
PHC: Primary health care
DASS-21: Depression, Anxiety and Stress Scale-21 Items
UHC: Universal health coverage

## References

[1] Zarei E, Ahmadi F, Sial MS, Hwang J, Thu PA, Usman SM (2019). Prevalence of burnout among primary health care staff and its predictors: a study in Iran. Int J Environ Res Public Health.

[2] Van Weel C, Kidd MR (2018). Why strengthening primary health care is essential to achieving universal health coverage. CMAJ.

[3] Halcomb E, Williams A, Ashley C, McInnes S, Stephen C, Calma K (2020). The support needs of Australian primary health care nurses during the COVID-19 pandemic. J Nurs Adm Manag.

[4] Chen Z, Zhou L, Lv H, Sun K, Guo H, Hu J (2021). Effect of healthcare system reforms on job satisfaction among village clinic doctors in China. Hum Resour Health.

[5] Deng J, Sun Y, Lei R, Guo Y, Liu J, Yang T (2019). Status of healthcare workers after comprehensive reform of urban public hospitals in Beijing, China: sustainable supply, psychological perception, and work outcomes. Hum Resour Health.

[6] Hatamizadeh M, Hosseini M, Bernstein C, Ranjbar H (2019). Health care reform in Iran: implications for nurses’ moral distress, patient rights, satisfaction and turnover intention. J Nurs Adm Manag.

[7] Cordioli DFC, Cordioli JR, Gazetta CE, Silva AGd, Lourenção LG (2019). Occupational stress and engagement in primary health care workers. Revista brasileira de enfermagem.

[8] Putri NK, Melania M, Fatmawati SMY, Lim YC (2023). How does the work-life balance impact stress on primary healthcare workers during the COVID-19 pandemic?. BMC Health Serv Res.

[9] Akova İ, Hasdemir Ö, Kiliç E (2021). Evaluation of the relationship between burnout, depression, anxiety, and stress levels of primary health-care workers (Center Anatolia). Alexandria J Med.

[10] Pulagam P, Satyanarayana PT (2021). Stress, anxiety, work-related burnout among primary health care worker: a community based cross sectional study in Kolar. J Family Med Prim Care.

[11] Zhen STE, Mohd TAMT, Ismail SMM, Woon GC, Chin TF, Meng OW (2020). Mental health status of healthcare workers in primary health clinics in Sepang. Malaysian J Psychiatry.

[12] Ferrari A, Somerville A, Baxter A, Norman R, Patten S, Vos T (2013). Global variation in the prevalence and incidence of major depressive disorder: a systematic review of the epidemiological literature. Psychol Med.

[13] World Health Organization. Depression and other common mental disorders: global health estimates. Geneva: World Health Organization. 2017;24. https://www.who.int/publications/i/item/depression-global-health-estimates.

[14] Salaton NF, Bulgiba A (2022). Depression, anxiety, and stress among frontline primary health care workers during the COVID-19 pandemic. Asia Pac J Public Health.

[15] Hajebi A, Abbasinejad M, Zafar M, Hajebi A, Taremian F (2022). Mental health, burnout, and job stressors among healthcare workers during the COVID-19 pandemic in Iran: a cross-sectional survey. Front Psychiatry.

[16] Al Lawati A, Al Ghafri T, Anwar H, Al Ajmi F, Al Hasani S, Chan M (2021). Depressive symptoms among primary healthcare workers during the novel SARS-CoV-2 coronavirus pandemic in the Muscat governorate. Prim Health Care Res Dev.

[17] Halcomb E, Fernandez R, Mursa R, Stephen C, Calma K, Ashley C (2022). Mental health, safety and support during COVID-19: a cross‐sectional study of primary health care nurses. J Nurs Adm Manag.

[18] Draucker CB, Rawl SM, Vode E, Carter-Harris L (2020). Integration through connecting in explanatory sequential mixed method studies. West J Nurs Res.

[19] Khademian F, Delavari S, Koohjani Z, Khademian Z (2021). An investigation of depression, anxiety, and stress and its relating factors during COVID-19 pandemic in Iran. BMC Public Health.

[20] Antony MM, Bieling PJ, Cox BJ, Enns MW, Swinson RP (1998). Psychometric properties of the 42-item and 21-item versions of the Depression anxiety stress scales in clinical groups and a community sample. Psychol Assess.

[21] Lovibond SH, Lovibond PF. Manual for the depression anxiety stress scales. Psychology Foundation of Australia; 1996.

[22] Lovibond PF, Lovibond, SHJBr, editors. therapy. The structure of negative emotional states: Comparison of the Depression Anxiety Stress Scales (DASS) with the Beck Depression and Anxiety Inventories. 1995;33(3):335 – 43. 10.1016/0005-7967(94)00075-u 7726811

[23] Asghari A, Saed F, Dibajnia P (2008). Psychometric properties of the Depression anxiety stress Scales-21 (DASS-21) in a non-clinical Iranian sample. Int J Psychol.

[24] Kakemam E, Navvabi E, Albelbeisi AH, Saeedikia F, Rouhi A, Majidi S (2022). Psychometric properties of the Persian version of depression anxiety stress Scale-21 items (DASS-21) in a sample of health professionals: a cross-sectional study. BMC Health Serv Res.

[25] Hennink M, Kaiser BN (2022). Sample sizes for saturation in qualitative research: a systematic review of empirical tests. Soc Sci Med.

[26] Polit D, Beck C (2020). Essentials of nursing research: appraising evidence for nursing practice.

[27] Havaei F, Astivia OLO, MacPhee M (2020). The impact of workplace violence on medical-surgical nurses’ health outcome: a moderated mediation model of work environment conditions and burnout using secondary data. Int J Nurs Stud.

[28] Havaei F, Ma A, Staempfli S, MacPhee M (2021). Nurses’ workplace conditions impacting their mental health during COVID-19: a cross-sectional survey study.

[29] World Health Organization. Workplace health promotion- Benefits. 2015.

[30] Ackah VA, Kwashie AA (2023). Exploring the sources of stress among operating theatre nurses in a Ghanaian teaching hospital. Int J Afr Nurs Sci.

[31] Ghavidel F, Fallahi-Khoshknab M, Molavynejad S, Zarea K (2019). The role of organizational factors in nurse burnout: experiences from Iranian nurses working in psychiatric wards. J Family Med Prim care.

[32] Kakemam E, Raeissi P, Raoofi S, Soltani A, Sokhanvar M, Visentin DC (2019). Occupational stress and associated risk factors among nurses: a cross-sectional study. Contemp Nurse.

[33] Tabrizi JS, Pourasghar F, Nikjoo RG (2017). Status of Iran’s primary health care system in terms of health systems control knobs: a review article. Iran J Public Health.

[34] Doshmangir L, Shirjang A, Assan A, Gordeev VS (2023). Iranian primary health care network: challenges and ways forward. Prim Health Care Res Dev.

[35] Nekoei Moghadam M, Amiresmaili M, Sadeghi V, Zeinalzadeh AH, Tupchi M, Parva S (2018). A qualitative study on human resources for primary health care in Iran. Int J Health Plann Manag.

[36] Johan S, Sarwar H, Majeed I (2017). To identify the causes of stress among nurses working in intensive care unit of Ittefaq Hospital Lahore. Int J Social Sci Manage.

[37] Smallwood N, Pascoe A, Karimi L, Bismark M, Willis K (2021). Occupational disruptions during the COVID-19 pandemic and their association with healthcare workers’ mental health. Int J Environ Res Public Health.

[38] Alyahya SA, Al-Mansour KA, Alkohaiz MA, Almalki MA. Association between role conflict and ambiguity and stress among nurses in primary health care centers in Saudi Arabia during the coronavirus disease 2019 pandemic: a cross-sectional study. Medicine. 2021;100(37). 10.1097/MD.0000000000027294 PMC8447988 34664892

[39] Kokoroko E, Sanda MA (2019). Effect of workload on job stress of Ghanaian OPD nurses: the role of coworker support. Saf Health work.

[40] Jin Y, Wang H, Wang D, Yuan B (2019). Job satisfaction of the primary healthcare providers with expanded roles in the context of health service integration in rural China: a cross-sectional mixed methods study. Hum Resour Health.

[41] Babamohamadi H, Davari H, Safari A-A, Alaei S, Pordanjani SR (2023). The association between workload and quality of work life of nurses taking care of patients with COVID-19. BMC Nurs.

[42] Feingold JH, Peccoralo L, Chan CC, Kaplan CA, Kaye-Kauderer H, Charney D (2021). Psychological impact of the COVID-19 pandemic on frontline health care workers during the pandemic surge in New York City. Chronic Stress.

[43] Skogsberg M, Jarl G, Matérne M (2022). Health care workers’ need for support from managers during the initial phase of the COVID-19 pandemic. BMC Health Serv Res.

[44] Vonderlin R, Müller G, Schmidt B, Biermann M, Kleindienst N, Bohus M (2021). Effectiveness of a mindfulness-and skill-based health-promoting leadership intervention on supervisor and employee levels: a quasi-experimental multisite field study. J Occup Health Psychol.

[45] Dietz C, Zacher H, Scheel T, Otto K, Rigotti T (2020). Leaders as role models: effects of leader presenteeism on employee presenteeism and sick leave. Work Stress.

[46] Bryan B, Gayed A, Milligan-Saville J, Madan I, Calvo R, Glozier N (2018). Managers’ response to mental health issues among their staff. Occup Med.

[47] Harms PD, Credé M, Tynan M, Leon M, Jeung W (2017). Leadership and stress: a meta-analytic review. Leadersh Q.

[48] Petrie K, Gayed A, Bryan BT, Deady M, Madan I, Savic A (2018). The importance of manager support for the mental health and well-being of ambulance personnel. PLoS ONE.

[49] Mathers CD, Loncar D (2006). Projections of global mortality and burden of disease from 2002 to 2030. PLoS Med.

[50] Løkke A-K (2022). Leadership and its influence on employee absenteeism: a qualitative review. Manag Decis.

